# ﻿*Diplaziumclivicolum* (Polypodiales, Athyriaceae), a new fern species from southern Yunnan, China

**DOI:** 10.3897/phytokeys.261.149078

**Published:** 2025-08-11

**Authors:** Hong-Jin Wei, Zheng-Yu Zuo

**Affiliations:** 1 Eastern China Conservation Centre for Wild Endangered Plant Resources, Shanghai Chenshan Botanical Garden, Shanghai, 201602, China Shanghai Chenshan Botanical Garden Shanghai China; 2 Center for Interdisciplinary Biodiversity Research & College of Forestry, Shandong Agricultural University, Tai’an, Shandong, 271018, China Shandong Agricultural University Shandong China; 3 Germplasm Bank of Wild Species, Kunming Institute of Botany, Chinese Academy of Sciences, Kunming, Yunnan, 650201, China Chinese Academy of Sciences Kunming China

**Keywords:** *Diplaziumincomptum*, *D.petiolare*, plastomes, section Anisogonium, sister species

## Abstract

A new species in Athyriaceae, *Diplaziumclivicolum* (section Anisogonium) is described and illustrated from southern Yunnan, China. *Diplaziumclivicolum* exhibits the greatest morphological similarity to *D.incomptum*, particularly in lamina division, but can be reliably differentiated, based on lamina width and lobe shape. Molecular phylogenetic analysis demonstrates that the new species is closely related to *D.petiolare*, despite low morphological concordance. This study focuses on comparing the morphological characteristics of these three species.

## ﻿Introduction

*Diplazium* SW. is one of the five most species-rich fern genera, each possessing over 300 species (PPG I 2016). In China, there are approximately 92 *Diplazium* species (Liu et al. 2024), with about half belonging to D.sect.Dolichostegia (W.M.Chu & Z.R.He) R.Wei, with section Anisogonium (W.M.Chu & Z.R.He) R.Wei, which contains about 12 species, exhibiting the second-largest species diversity, based on the new sectional classification from Wei et al. (2025).

Xishuangbanna Dai Autonomous Prefecture is located at the southernmost tip of Yunnan Province and is home to the largest tropical rainforest reserve of China, with at least 400 fern species, around 20 of which belong to *Diplazium* (Zuo et al. unpublished data). In 2024, the authors collected an unknown *Diplazium* plant when undertaking a preliminary survey of fern resources within this region. This plant is morphologically similar to *D.incomptum* Tagawa in having pinnatipartite pinnae, linear sori and ascending or erect rhizomes. However, subsequent molecular phylogenetic analysis revealed that the plant is more closely related to *D.petiolare* C.Presl, despite sharing fewer morphological similarities. We name the unknown plant *D.clivicolum* and describe it below.

## ﻿Materials and methods

### ﻿Molecular phylogeny

The molecular phylogenetic analyses were conducted using whole plastomes. Total DNA of the newly-discovered taxon was extracted from leaf fragments preserved in silica gel following the CTAB method (Doyle and Doyle 1987). Library construction, Illumina sequencing and plastid DNA assembly followed Zuo et al. (2025), with *Diplaziumdilatatum* Blume (NC_035850) as the reference. To reconstruct the phylogenetic relationships of *D.clivicolum*, we retrieved 174 *Diplazium* plastomes from NCBI (see Suppl. material 1, mostly from Wei et al. (2025)), including morphologically similar species such as *D.incomptum*. Amongst these, 63 species originated from China, representing approximately 68% of all Chinese *Diplazium* species listed in Liu et al. (2024). The final plastome matrix was comprised of 175 *Diplazium* samples (174 species) and 15 outgroups, using MAFFT v.7.017 (Katoh et al. 2002) and Geneious 9.1.4 (Kearse et al. 2012) for alignment and correction of the matrices. Maximum Likelihood (ML) analyses were performed utilising IQ-TREE 1.6.12 (Nguyen et al. 2015) with the GTR+R6 model and 1000 ultrafast bootstrap replicates. Bayesian Inference (BI) analysis was conducted using MrBayes 3.2.6 (Ronquist et al. 2012) for ten million generations with sampling every 1000 generations, employing two Markov Chain Monte Carlo (MCMC) runs. The first 25% of trees were discarded as burn-in.

### ﻿Morphological comparison

Species for comparison included the unknown taxon, its morphologically most similar species *D.incomptum* and phylogenetically most closely-related species *D.petiolare*, as revealed by our molecular analysis. Their morphological attributes were analysed based on previous research (e.g. Presl 1851; He and Kato 2013; Ebihara 2017; Wei et al. 2025) and examinations of physical and digitised specimens. Approximately 40 scales from the stipes and fiddlehead, along with all spores from an indusium of the unknown taxon, were observed and photographed using two different models of stereo-microscopes (Olympus SZX16 and ZEISS AX10). Physical specimens of the related species in CSH and KUN were examined. Additionally, digitalised specimens were observed from databases including CVH (https://www.cvh.ac.cn/), Digital Taiwan (https://digitalarchives.tw/), GBIF (https://www.gbif.org/), JSTOR (https://plants.jstor.org/) and Plants of TAIWAN (https://tai2.ntu.edu.tw/index.php).

## ﻿Results and discussion

**Figure 1.**
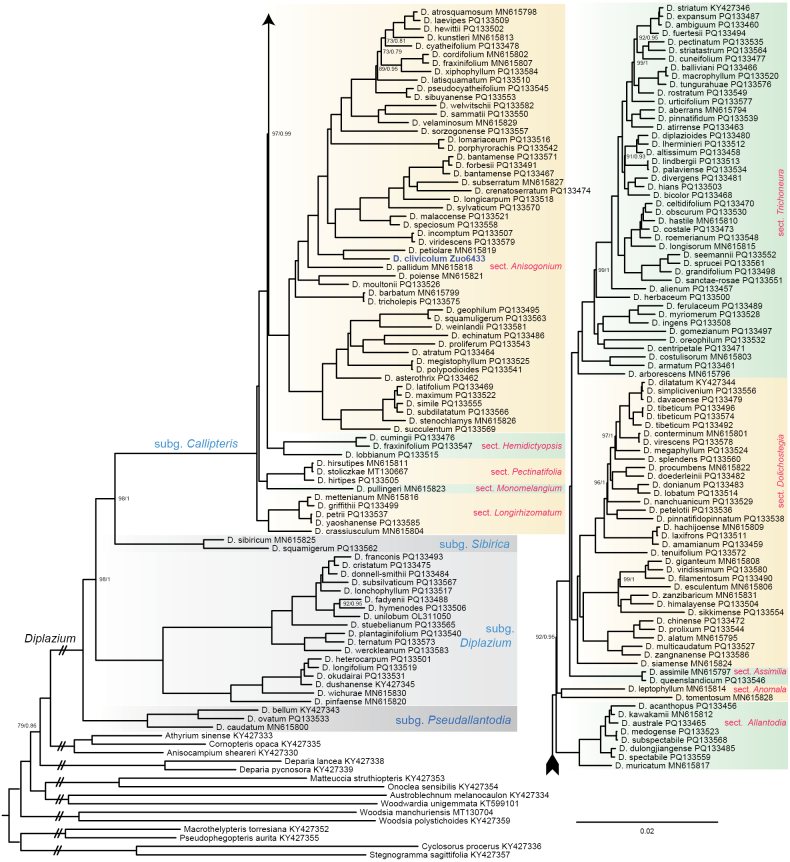
The Maximum Likelihood (ML) phylogram of *Diplazium* based on plastomes. Nodes without support values indicate that ultrafast bootstrap values (UFBoot) are 100% and Bayesian posterior probabilities (PP) are 1.0.

*Diplaziumclivicolum* was resolved as a sister species to *D.petiolare* (Fig. 1), within section Anisogonium which is native to Sumatra, Borneo and the Philippines. Wei et al. (2025) demonstrated that, based on current data, *D.petiolare* appears to be a single lineage with no sister species. However, *D.clivicolum* is currently known to be found exclusively in tropical China, at a distance exceeding 2000 km from the known range of *D.petiolare*. Our phylogeny suggests that these two allopatric species likely derived from a common ancestor following geographic isolation.

**Figure 2.**
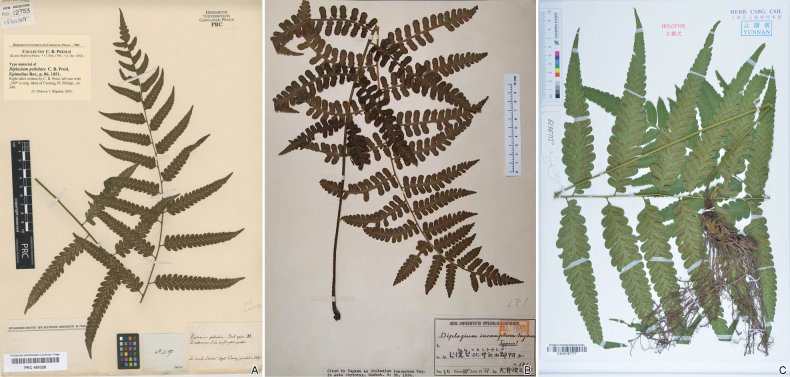
Types of *Diplaziumpetiolare* (A), *D.incomptum* (B) and *D.clivicolum* (C).

In morphology, *D.clivicolum* exhibits many divergences from *D.petiolare* rather than similarities. The similarities between them are limited to a few characteristics, for instance, rhizome form and sori length. *Diplaziumpetiolare* features lanceolate or narrowly triangular lamina, pinnatilobate pinnae, hispid rachis and costae, oblique-truncate lobe apices (Fig. 2A), all of which are significantly different from those found in *D.clivicolum*. On the contrary, *D.clivicolum* shares more similarities with *D.incomptum*, including the erect rhizome, lanceolate scales on rhizome and stipe, sometimes ovate lamina, pinnatipartite or pinnatisect pinnae, subglabrous rachis and costae, as well as medial sori on veinlets (Fig. 2B). However, *D.clivicolum* is distinguished by marked differences, such as longer rhizome scales, broader lamina, suddenly constricted lamina apex, oblong-obovate lobes without obviously undulate margins and longer sori (Fig. 2C).

Interestingly, according to He and Kato (2013) and Wei et al. (2025), the vast majority of *Diplazium* species distributed in China exhibit sister species in relatively close proximity. While the occurrence of a sister species for *D.clivicolum* at such a geographically distant location may be the result of long-distance dispersal or other biogeographic factors, it warrants further investigation given the general pattern observed in the genus. We hypothesize that future investigations may reveal additional closely-related species of this taxon in geographically proximate regions.

### ﻿Taxonomic treatment

#### ﻿ Diplazium clivicolum

Taxon classificationPlantaePolypodialesAthyriaceae

H.J.Wei & Z.Y.Zuo sp. nov.

65BAAB5E-D9F1-5F3A-A1ED-8D2387BB82F5

urn:lsid:ipni.org:names:77367033-1

Figs 3 , 4

##### Type.

China • Yunnan: Mengla County, Yaoqu Yao Ethnic Town, Sharen Village, on slope in evergreen broad-leaved forest, 21°48'N, 101°35'E, elev. 930 m, 31 July 2024, *She-Lang Jin & Zheng-Yu Zuo JSL9679* (holotype: CSH0197777!; isotypes: CSH!, IBK!, KUN!, PE!).

##### Diagnosis.

*Diplaziumclivicolum* morphologically resembles *D.incomptum* in terms of rhizome type, lamina division, serration of lobe edge, venation pattern and sori position and shape, but differs by having broader lamina, contracted lamina apex, asymmetric pinna base, oblong-obovate and sometimes imbricate lobes and longer sori.

##### Description.

***Plants*** evergreen, medium-sized. ***Rhizome*** ascending to erect, with dense, black-brown, stiff, fleshy, long, thick roots, apex densely scaly; ***scales*** brown or dark brown, lanceolate, seldom broadly lanceolate, 5–8 × 1–2 mm, membranous, margins slightly denticulate or nearly entire, with narrow, fragmented black edges. ***Fronds*** caespitose, 86–106 cm; ***stipe*** (37–)43–53 cm, 3.5–6 mm in diam. at base, 2–3 mm in diam. near middle, base black-brown, densely covered with scales similar to those on rhizome, above base stramineous, sparsely covered with adpressed, lanceolate scales, upwards stramineous, subglabrous, shallowly grooved adaxially; ***lamina*** pinnate-pinnatipartite or pinnate-pinnatisect near base, ovate, 45–54 × 38–51 cm, apex somewhat abruptly narrowed and elongated acute; ***pinnae*** 9–10 pairs, lower pairs 16–26 × 3–4.3 cm, broadest at middle or below, slightly narrowed towards base, alternate or subopposite, ascending, lanceolate, stalked, pinnatilobate to pinnatifid, base asymmetrical with basal adaxial and abaxial lobes alternate, apex long acuminate; ***basal pair of pinnae*** slightly shortened, pinnatipartite, 16–20 cm, with stalk 4–11 mm; ***second pair of pinnae*** pinnatipartite or pinnatisect to costa, 20–26 cm, with stalk 3.5–7 mm, upper pinnae gradually reduced below cuspidate apex; ***pinna lobes*** often up to 12 pairs, ascending, approximate, contiguous or imbricate, oblong to oblong-obovate, 12–25 × 7–11 mm, base often narrowed and cuneate when lamina fully developed, adnate to narrow wings, margins notched, repand, repand-toothed or crenate, apex rounded or rounded-truncate, basal pair of pinna lobes on lower pinnae anadromous, often abruptly reduced, sometimes completely free, narrowly or broadly adnate; ***veins*** pinnate, prominent abaxially, visible adaxially, veinlets 5–7 pairs, oblique, simple, seldom forked. ***Lamina*** thickly herbaceous or papery when dry, green adaxially, yellow-green abaxially, glabrous on both surfaces; ***rachis and costae*** stramineous, adaxially shallowly grooved, with abundant squarrose-glandular papillae in grooves, abaxially subglabrous along rachis and with sparse brown long appressed articulate hairs along costae. ***Sori*** linear, medial, often up to 9 mm, seldom up to 10 mm, 5–7 pairs per lobe, single or double on acroscopic basal veinlet, oblique, from near mid-rib to near margin; ***indusia*** brown, linear, membranous, entire, persistent. ***Spores*** light brown, reniform to phaseoliform, 22–32 μm in polar axis,40–56 μm in equatorial axis, perispore with a few long and low folds, forming a rugate surface, 64 per sporangium.

**Figure 3.**
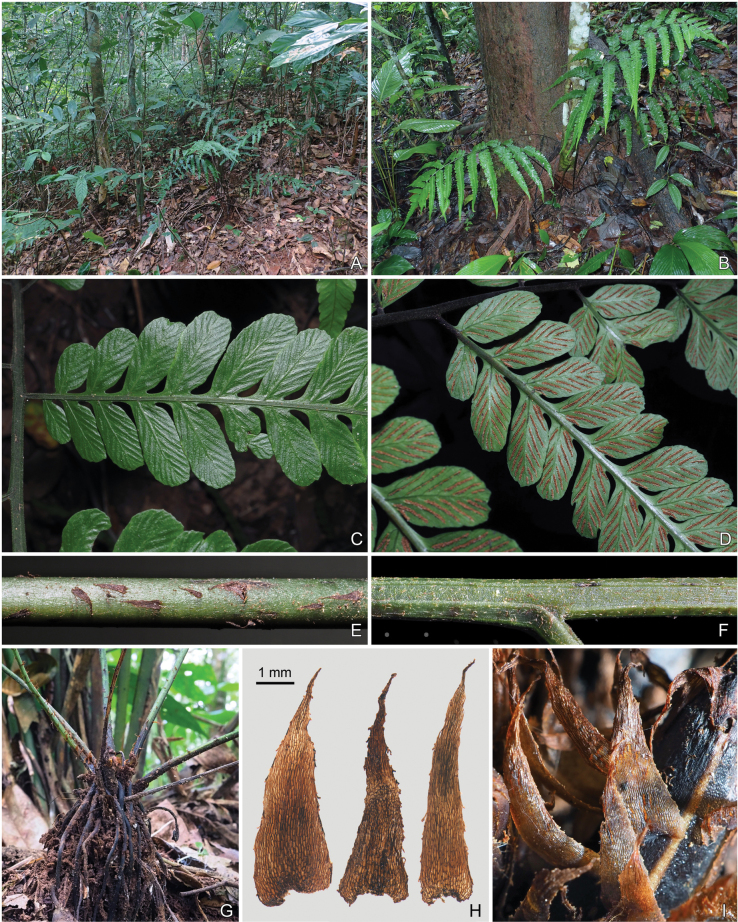
*Diplaziumclivicolum*. A. Habitat; B. Habit; C. Adaxial view of portion of pinna; D. Abaxial view of portion of pinnae; E. Portion above base of stipe; F. Adaxial view of lower portion of pinna stalk with portion of rachis; G. Rhizome with lower portion of stipes; H. Scales from stipe base (left and middle) and 2 cm-tall fiddlehead (right); I. Stipe base exhibiting scales. Photographed by Hong-Jin Wei.

##### Notes.

Characterised by the decumbent or erect rhizome bearing scales, pinnate-pinnatipartite fronds, alternate pinnae with stalks, but lacking auricles at the acroscopic bases, free veins and sori longer than two-thirds the length of the veins. *Diplaziumclivicolum* should be classified as a member of section Anisogonium on the basis of the key descriptions of Wei et al. (2025) and supported by our phylogenetic analysis.

**Figure 4.**
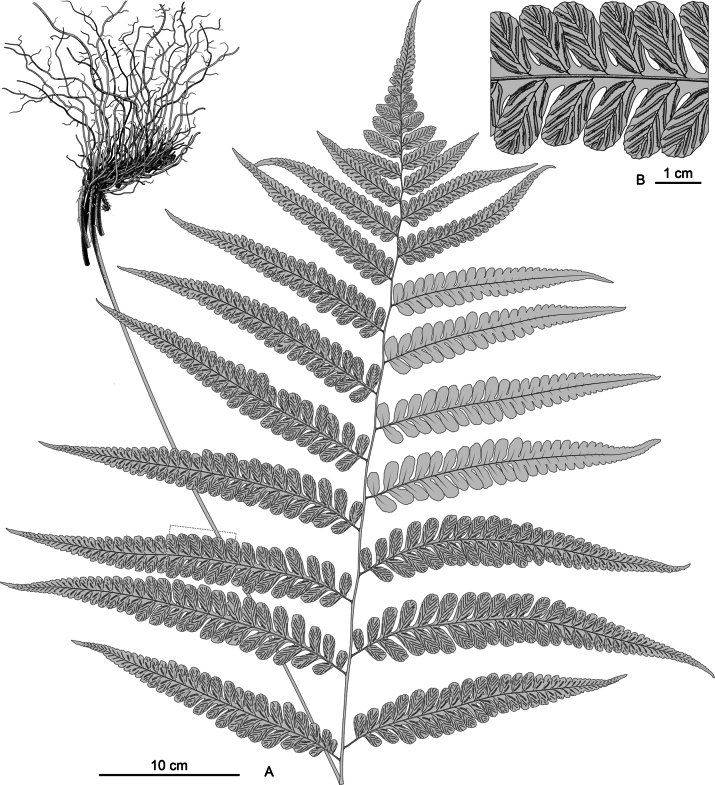
*Diplaziumclivicolum*. A. Habit; B. Portion of pinna showing veins and sori. Illustrated by Hong-Jin Wei.

##### Geographical distribution.

**Figure 5.**
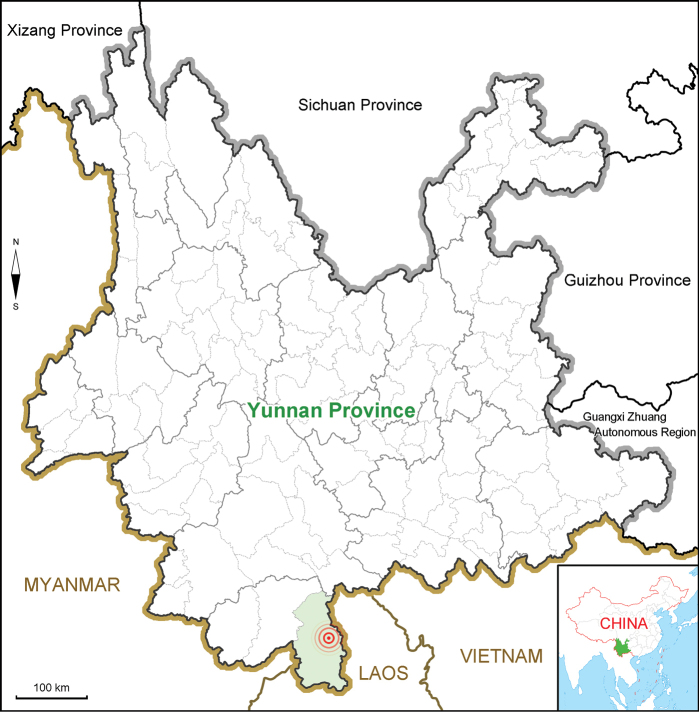
Geographical distributions of *Diplaziumclivicolum* (center of rings) in Yunnan Province, China.

Currently only found at the type locality, belonging to Xishuangbanna National Nature Reserve (Fig. 5).

##### Ecology and conservation assessment.

One population of approximately 30–50 individuals was discovered on a hillside within an evergreen broad-leaved forest, at an elevation of roughly 910–930 m. However, this habitat is just located at the Reserve boundary adjacent to an extensive farmland and close to a village, placing it at ongoing risk of destruction. In accordance with IUCN Criteria (IUCN 2012), the new species is temporarily classified as critically endangered. Given the abundance of similar ecological niches in this area, it is hoped future surveys will reveal more habitat locations.

##### Etymology.

The specific name is derived from the Latin term “*clivicolum*”, which means “*dwelling on a slope or hillside*”, referring to its habitat.

##### Chinese name.

坡生短肠蕨 (pō shēng duǎn cháng jué)

##### Paratypes.

Same place as the holotype, 31 Jul 2024 *Zheng-Yu Zuo Zuo6433* (KUN!)·ibid., 31 Oct 2024, *She-Lang Jin, Zheng-Yu Zuo & Rui Zhang JSL10125* (CSH!)·ibid., *Zheng-Yu Zuo Zuo7161* (KUN!)

## Supplementary Material

XML Treatment for Diplazium clivicolum

## ﻿Additional information

### Use of AI

No use of AI was reported.

### Author ORCIDs

Hong-Jin Wei https://orcid.org/0000-0002-3679-6683

Zheng-Yu Zuo https://orcid.org/0000-0002-8334-6132

### Data availability

All of the data that support the findings of this study are available in the main text or Supplementary Information.

## Supplementary materials

Supplementary material 1 Information of voucher specimens and GenBank accession numbers of plastomes in this study Data type xlsx https://binary.pensoft.net/file/1392434This dataset is made available under the Open Database License (http://opendatacommons.org/licenses/odbl/1.0/). The Open Database License (ODbL) is a license agreement intended to allow users to freely share, modify, and use this Dataset while maintaining this same freedom for others, provided that the original source and author(s) are credited.Hong-Jin Wei, Zheng-Yu Zuo
